# Using sensory discrimination in a foraging-style task to evaluate human upper-limb sensorimotor performance

**DOI:** 10.1038/s41598-019-42086-0

**Published:** 2019-04-09

**Authors:** Dylan T. Beckler, Zachary C. Thumser, Jonathon S. Schofield, Paul D. Marasco

**Affiliations:** 1 Laboratory for Bionic Integration, Lerner Research Institute, Department of Biomedical Engineering, Cleveland Clinic, Cleveland, OH USA; 2 Research Service, Louis Stokes Cleveland Department of Veterans Affairs Medical Center, Cleveland, OH USA; 3 Advanced Platform Technology Center of Excellence, Louis Stokes Cleveland Department of Veterans Affairs Medical Center, Cleveland, OH USA

## Abstract

Object stiffness discrimination is fundamental to shaping the way we interact with our environment. Investigating the sensorimotor mechanisms underpinning stiffness discrimination may help further our understanding of healthy and sensory-impaired upper limb function. We developed a metric that leverages sensory discrimination techniques and a foraging-based analysis to characterize participant accuracy and discrimination processes of sensorimotor control. Our metric required searching and discriminating two variants of test-object: rubber blocks and spring cells, which emphasized cutaneous-force and proprioceptive feedback, respectively. We measured the number of test-objects handled, selection accuracy, and foraging duration. These values were used to derive six indicators of performance. We observed higher discrimination accuracies, with quicker search and handling durations, for blocks compared to spring cells. Correlative analyses of accuracy, error rates, and foraging times suggested that the block and spring variants were, in fact, unique sensory tasks. These results provide evidence that our metric is sensitive to the contributions of sensory feedback, motor control, and task performance strategy, and will likely be effective in further characterizing the impact of sensory feedback on motor control in healthy and sensory-impaired populations.

## Introduction

The ability to detect an object’s compliance plays a fundamental role in shaping the exploration and manipulation of that object within its environment. The resulting sensory information drives the identification, classification, or discrimination of the object and directly influences the motor interactions. For example, when an object of high compliance is sensed by the fingers and hand, it may be an indication of fragility, and as such the contact forces imparted by the hand will be precisely controlled to avoid crushing or damaging the object^1^. Here, sensory feedback informs motor output, creating a physiological closed-loop control system. These sensorimotor mechanisms underpin motor-error correction and adjustment to perturbation during object manipulation^2^.

This interrelationship between sensation and motor control is particularly evident in sensory-impaired populations, often presenting as significant motor deficits; an active area of study in those affected by stroke^3–5^, multiple sclerosis^6^, and cerebral palsy^6–9^. Inversely, in these populations the presence of sensation, or improved sensation through intervention, has been observed to improve motor function^5–8,10^. Beyond observing affected populations, conventional sensory-assessment tools typically evaluate motor function and sensation independently, providing limited quantitative insight in characterizing the impact of sensation on motor control. This inadequacy creates challenges in determining the success of sensory-related interventions as the effect of sensation on overall function cannot be directly evaluated. This has particularly relevant implications in determining the influence of sensation on function in a number of sensory-impacted populations such as neural-machine interfaces including users of advanced sensate limb prostheses, and brain computer interfaces. Other populations of interest include stroke, hand transplantation, and upper limb involved spinal cord injury. Current assessments often use discrimination paradigms to evaluate one’s ability to detect changes exclusively in sensation^11–15^. In this study, we combine a sensory discrimination paradigm and a ‘search-and-select’ style foraging task to evaluate the effectiveness of motor and sensory strategies.

Many of the activities we perform as humans may be considered nothing more than the successive execution of search-and-acquisition tasks. As we go through our day, scouring the refrigerator for food, searching for our keys, looking for a parking spot, or finding a table in the cafeteria, we engage in goal-motivated search-directed behaviors. The same mathematics that describe animals foraging, such as lions searching and hunting for prey on the savannah^16^, have applicability to a vast range of goal-oriented search-and-acquisition type human behaviors. An early investigation of these fundamental mathematical relationships was presented in Holling’s seminal 1959 *disc equation* paper^17^. Holling investigated how parameters such as prey density, forager search rate, and prey handling rate affected the overall rate of prey capture. Additionally, Holling developed several of the axioms that formed the foundations of modern optimal foraging theory (OFT), such as the mutual exclusivity of the time spent searching for prey versus consuming it^18^.

Modern OFT is a collection of mathematical models that describe how biological foragers search for, acquire, and consume food. The core concept of these well validated models is that organisms inherently adopt strategies, movement patterns, and prey types that maximize their long-term rate of gain, as natural selection favors these behaviors^19–22^. The net energy gained from a prey item is the difference between its caloric content and the energy expenditure required to capture and consume the prey^22^. The time spent foraging is generally divided into two phases: a search phase where the forager spends time locating prey, and a handling phase where the forager spends time acquiring (e.g., chasing, hunting, and gathering) and consuming the prey. Thus, rate of gain is mathematically defined as the net energy gained divided by the total time spent foraging, and is used to describe the relative value of prey items and/or forager behavior. *Profitability* is a measure derived by dividing net caloric energy by handling time. This value provides perspective into foraging strategy, for instance when a potential prey item is encountered, the decision to consume it or continue to forage for a higher reward item is influenced by the profitability of prey items in the environment. Long-term rate of gain and profitability have helped provide insight into biological decision-making in a broad range of foraging species, such as bacteria^23^, insects^24,25^, fish^26^, birds^20,27,28^, and mammals^25,29,30^.

There is flexibility inherent to this mathematical approach. For instance, the general concept of ‘caloric energy’ can be interchanged with ‘task performance’^31^, which then provides the mathematical foundation for OFT to describe biological, micro-economic, and “all human choice-making” phenomena^32,33^. In this context, the utility of OFT has been vastly expanded to describe numerous biological and human behaviors including: navigation^34^, information gathering^32,35^, tool use^36,37^, scholastic performance^38^, domestic burglary^39^, and design-and-analysis optimization^31^.

Combining sensory discrimination with a foraging task would closely align with the well-established variant of OFT known as the cryptic prey model. Traditionally, the cryptic prey model describes the situation when prey is not readily identifiable as food and must be discriminated by the forager^40^. Therefore, the forager must spend time to recognize the prey item, a quantity defined as recognition time^40,41^. Conventionally, this model has been used to study forager decision-making processes with regard to discrimination of prey species^42^, prey size^20,42^, numerical distribution^43^, and non-food items^44^. This model provides appropriate evaluation tools for human sensorimotor performance as it inherently accounts for discrimination and decision-making activities. In fact, the aforementioned Holling disk equation was initially developed using a tactile-based sensory task, as human participants were instructed to forage for sandpaper discs while blindfolded^17^. Interestingly, Holling keyed into a distinguishing facet of the cryptic prey model, 20 years before the model would be explicitly defined^41^; when he asked participants to locate the sandpaper discs by tapping a pencil, he noted a moment of hesitation before each disc was handled. He termed this time period, “identification time”, a concept which would later be refined by Hughes as “recognition time”^41^. Despite OFT’s foundation and demonstrated applicability in sensory tasks, it is seldom used as a platform for investigating psychophysics and sensory processes *per se*. Here, we develop a sensorimotor assessment toolset that utilizes a video analysis, similar to cryptic prey video analyses used in OFT^20,21^, to quantify participant speed and performance in a tactile and proprioceptive foraging-style search-and-acquisition task.

## Methods

To maximize the ease of implementation, and the potential utility of our measures, we developed our assessment task within the context of a set of pre-defined goals: (1) minimal rules and instructions, such that participants’ performance strategies are unrestricted, (2) sensitivity to the participants’ sensorimotor strategies, (3) sensitivity to the effects of sensory feedback by specifically forcing discrimination judgments informed through cutaneous or proprioceptive information, (4) the elimination of ceiling effects, providing the framework for potential comparison of multiple populations and interventions, and (5) multiple outcome scores rather than a single “good or bad”, or completion time score, allowing researchers a more in-depth examination of how participants use their sensory feedback and possible trade-offs they may be making.

### Participants

A cohort of fifteen able-bodied adults was recruited (11 female, 4 male, 13 right-handed, 2 left-handed, average age 28 years, age range 23–48 years). Participants reported no deficits in the mobility and sensation of their upper limbs. Each participant completed testing with their dominant hand. Research ethics approval was received through the Institutional Review Boards for the Cleveland Clinic and Department of Navy Human Research Protection Program, and all research was performed in accordance with these guidelines and regulations. All participants provided informed consent prior to participating in this study. Participants were naïve to the specific aims of the experiment, but were informed that they would be discriminating objects of varying stiffness, and that speed and accuracy would be measured. Two experiments were performed, *the block test*, in which polyurethane rubber blocks were manipulated, and *the spring test* in which custom-made spring cells were manipulated.

### The block test

#### Experimental setup

Our goal was to develop a functional test that was inexpensive, simple to implement, and in a format that was understandable and accessible to clinicians. This necessitated designing the format of the test with reproducible manufacturing and readily available materials. The block test used polyurethane rubber blocks (25.4 mm cubes) of different stiffnesses. Durometers of 40A, 60A, and 80A were selected to provide compressibility under physiological grip forces without sacrificing durability. Additionally, blocks in this range of durometers were similar in appearance and surface texture, minimizing the ability for participants to indirectly discriminate block stiffness through vision or roughness.

The distribution of durometers was based on findings by Srinivasan and LaMotte, who found that under “almost natural conditions”, participants can distinguish changes as small as 12% difference in the compliance of surface-deformable objects^1^. Because our rubber blocks were obtained based on shore durometer, stiffnesses for our materials were calculated based on empirically derived relationships between shore durometer and Young’s modulus^45^, and the known dimensions of our rubber blocks. We found that the percent difference (absolute difference divided by average) of stiffness (applied force divided by displacement) increments used in our study were 7 to 8 times greater, assuring that participants would perform above guess-based chance.

**Figure 1:**
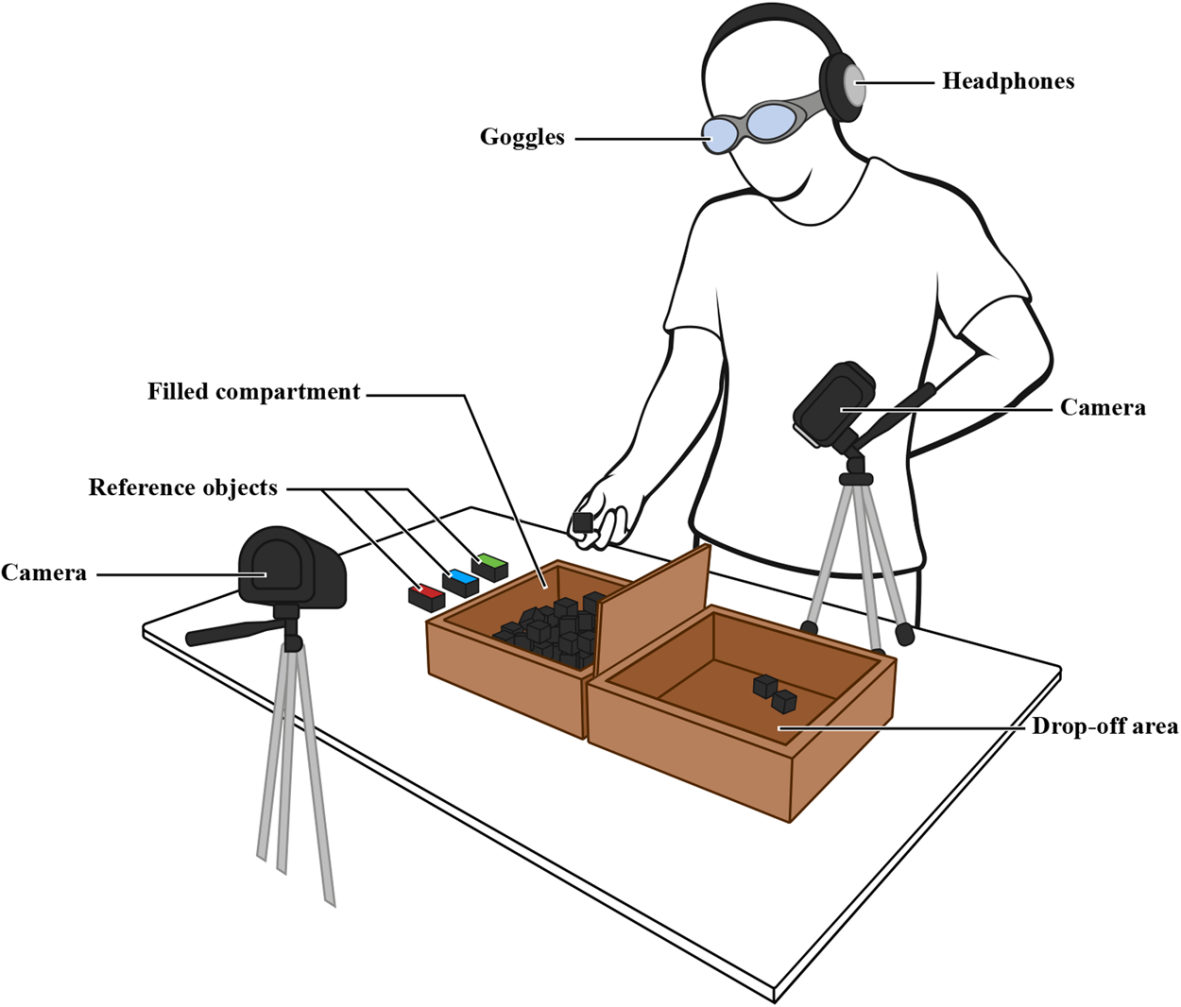
Schematic of the experimental setup. A two-compartment Box-and-Block-style setup had one compartment filled with polyurethane rubber blocks that the participant would discriminate, transfer over the center partition, and release in the drop-off area. The participant wore frosted goggles and noise cancelling headphones, and two digital recorders captured video data.

The experimental setup for the discrimination task (Fig. 1) was presented in a partitioned space to divide the *searchable* test-objects from the *selected* test-objects. In this instance we utilized the box from a standard two-compartment Box & Block test (Sammons Preston Inc., Boiling Brook, Il, USA)^46^ because it is a widely used and familiar format in standardized validated tests of upper limb motor performance^46,47^. However, in principle, any partition (e.g., a painted line or strip of tape) is sufficient for the purpose of this test.

One compartment was filled with a mixture of twenty each of 40A durometer (soft), 60A durometer (medium), and 80A durometer (hard) polyurethane blocks (60 blocks in total). The box was placed on a 0.75 m tall table, with the filled compartment on the same side as the participant’s dominant hand. Next to the filled compartment, we placed three reference blocks: one soft, one medium, and one hard, with the stiffnesses clearly labelled (“soft”, “medium”, or “hard”) on each block with colored adhesive tape. Two digital video recorders captured the testing area which included the participant’s testing hand, the filled compartment of the box, the empty compartment, and the reference blocks. Video data were captured at 30 frames per second.

During the experiment, participants wore disposable earplugs, noise cancelling headphones, and frosted goggles. Gray noise was played through the headphones to reduce possible auditory cues from the blocks being manipulated or dropped during testing. The frosted goggles were made from panoramic safety glasses which were finely misted with clear plastic paint. These goggles reduced visual acuity roughly by a factor of 3, thereby mitigating possible visual cues (such as material compressibility or sheen), while still allowing the blocks to be visually located within the experimental setup.

#### Experimental procedure

Participants stood in front of the two-compartment box. They were informed that the box contained soft, medium, and hard blocks and that they would be searching for either soft or hard blocks. Each trial began with the participant resting their testing hand on the table, and the investigator would indicate which stiffness the participant needed to search for (either soft or hard) by tapping the corresponding reference block. The participant would then search for five blocks of the target stiffness, one at a time, and transfer them over the center partition and into the other compartment of the box. When five blocks were successfully transferred, the trial was complete. The number of correct blocks transferred was reported to the participant, and the setup was reset for the next trial.

The quantity of five target blocks for each trial (out of a possible 20 target blocks) was selected to avoid significant depletion effects. Each time the participant grabbed a block, there was some probability that it would be a target block, based on the total proportion of correct blocks in the container. As the participant removed correct (or incorrect) blocks from the container the probability of encountering a correct block would decrease (or increase). For example, the probability of a participant encountering a correct block ranged from 33% at the beginning of a trial (20 correct blocks out of 60 blocks total) to a worst case scenario of 28.6% at the end of a trial if the first four blocks selected were all correct (16 correct blocks remaining out of 56 blocks total).

Participants were informed that speed and accuracy were both being measured, and that they should be as fast and accurate as possible. Additionally, they would periodically perform a baseline trial where they would not search for a particular stiffness, but instead, quickly transfer any five blocks into the other compartment. We explained that the investigator would tap the medium reference block when they should do a baseline trial. The baseline trials served to capture the time required to simply transfer a block to the empty compartment without a decision about stiffness (no discrimination).

At the beginning of the session, each participant completed six trials of practice: two soft, two hard, and two baseline trials. It was explained to the participants that the practice trials were not timed and that they should focus on resolving the differences between the stiffnesses. After completing the practice trials, participants completed 10 rounds of testing, where each round consisted of one soft, one hard, and one baseline trial in a pre-determined randomized order. In total, each participant sorted 50 blocks during soft trials, 50 during hard trials, and 50 during baseline trials, for a total of 150. Participants were given a rest of at least one minute after the third and sixth rounds of testing. Testing took approximately 30 to 45 minutes to complete, depending on the speed of the participant.

### The spring test

#### Experimental setup

**Figure 2:**
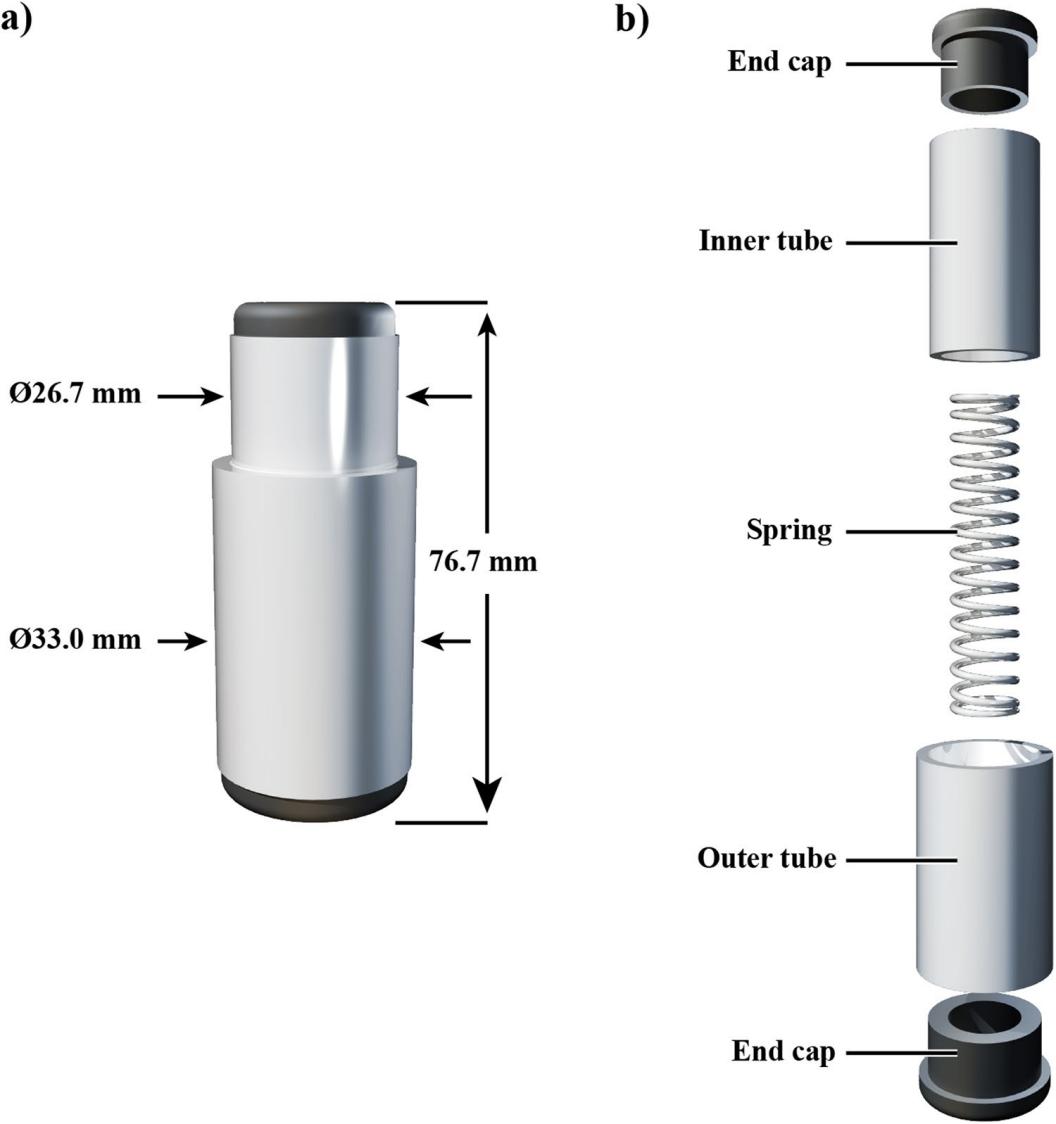
Spring cell design. (**a**) The overall dimensions of the assembled spring cells detailing diameters of the inner tube and outer tube, as well as the overall height. (**b**) An exploded assembly view of the spring cell components detailing the end caps, inner tube, outer tube, and spring.

The spring test setup was identical to the block test setup, except that the blocks were replaced with spring cells (Fig. 2). The spring cells provided large displacements when squeezed, thereby requiring a higher dependence on proprioceptive information to discriminate relative to the block test, which largely required cutaneous pressure information^1^. Each spring cell was constructed from two 38.1 mm-long telescoping aluminum tubes; the outer tube had 31.8 mm-outer diameter and the inner tube had 25.4 mm-outer diameter (Fig. 2). A 76.2 mm-long spring was epoxied to two opposing press-fit end caps, such that squeezing the telescoping tubes into each other compressed the spring along its longitudinal axis, providing approximately 25.4 mm of compression. Polytetrafluoroethylene (PTFE) tape was applied to the interior surface of the outer tube to reduce stiction and play in the construction. Three stiffnesses of springs were used, 1.4 N/mm, 2.1 N/mm, and 2.6 N/mm. Due to the size of the spring cells relative to the size of testing area, only 12 of each stiffness was used during testing (36 spring cells in total).

#### Experimental procedure

The spring test used the same procedure as the block test, with specific differences. The spring cells could conceivably be handled with at least two different methods: a cylinder grip where compression was done with the thumb, such as the way a joystick may be held while pressing a button located at its top; and a tripod grip where the index and/or middle finger opposed the thumb to squeeze the spring cell. We did not demonstrate any method to the participants or instruct them to use any specific grip, but after the practice trials, participants were instructed to continue using their selected grasping and squeezing strategy for the entirety of the test.

We instructed participants to sort four spring cells per trial (rather than the five sorted in the block test), because fewer spring cells were used, and we wanted to keep depletion effects similar between the two tasks (the probability of grabbing a correct spring cell by chance ranged from 33% to 27.3%). However, the number of trials was increased to 13 to compensate for the reduced number of springs discriminated per trial. In total, each participant discriminated 52 soft, 52 hard, and 52 baseline spring cells during testing.

### Scoring procedure

**Figure 3:**
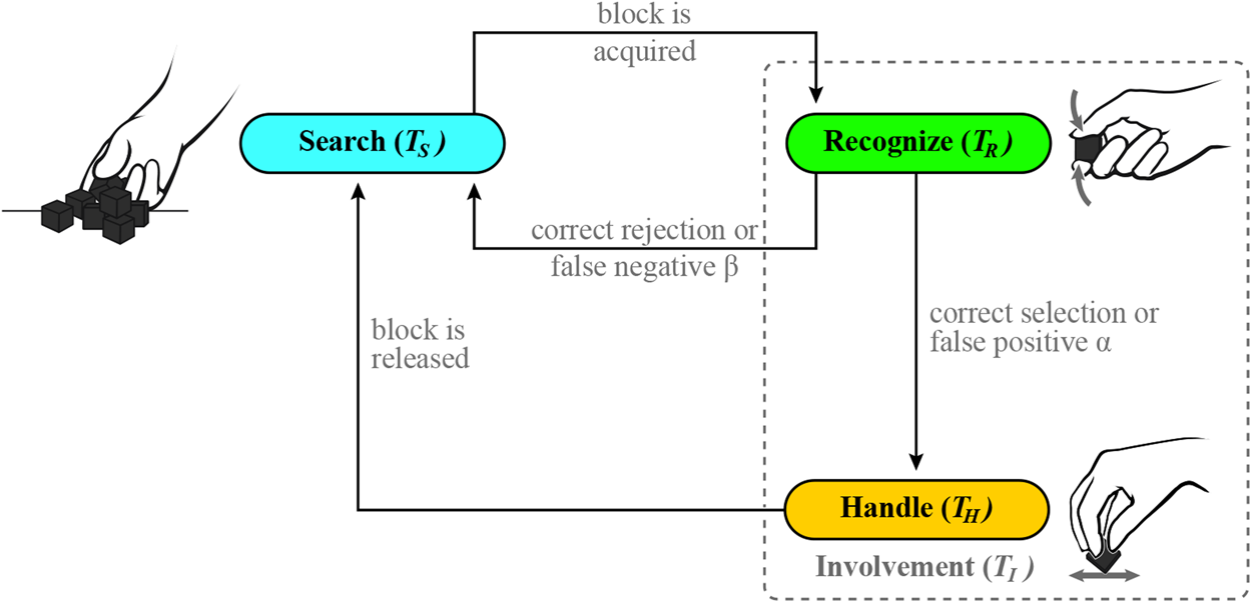
Foraging cycle. A breakdown of the foraging phases and time outcome measures extracted from review of experimental video footage. Where *T*_*S*_, *T*_*R*_, *T*_*H*_, and *T*_*I*_ denote *search time*, *recognition time*, *handling time*, and *involvement time*, respectively. The gray dashed box represents the time periods during testing that defined *involvement time*.

The block and spring tests were scored individually, post-experiment, via video analysis, according to the same protocol. For each participant, the video footage from both cameras was synchronized, and recordings were viewed in a media player which allowed forward and backward frame-by-frame scrubbing. We used a video analysis procedure that reflected video analyses used in cryptic foraging studies^20,21^ to extract four key time measures per trial from the video footage (Fig. 3): *search time* (*T*_*s*_), the time spent searching for a to-be-selected block or spring cell (referred to herein as test-objects); *involvement time* (*T*_*I*_), the time spent interacting with the to-be-selected test-object; *handling time* (*T*_*H*_), the time spent transferring the selected test-object over the center partition and into the empty compartment of the box; and *recognition time* (*T*_*R*_), the time spent extracting sensory information to inform decision making. Additionally, we documented the number of test-objects that the participants manipulated prior to making a selection. The start of each foraging cycle was explicitly defined as the moment a participant’s tested hand broke contact with the table as it was raised from the start position. This marked the start of *search time*, and was captured from the video footage timestamp. *Search time* ended when the participant first made contact with the test-object that they would ultimately select. This time also marked the beginning of *involvement time*. *Involvement* included all of the time that the participant spent interacting with the to-be-selected test-object, and ended when the participant transferred and released that test-object. The release marked the conclusion of a single foraging cycle and the start of the next one.

*Recognition time* represented the interval in which participants squeezed a to-be-selected object and interpreted the sensory information to inform their selection. The derivation of this value was dependent on the baseline trials that participants performed throughout the testing. As the baseline trials did not require a discrimination decision to be made, they were assumed to purely characterize the time required to grasp and transfer a test-object (defined as *handling time*). Therefore, the difference between the experimentally measured *involvement time* and the *handling time* quantified the time spent making a decision (*recognition time*). These values are shown formulaically in Eqn. 1,

1 $${T}_{R}={T}_{I}-{T}_{H}$$

where *T*_*R*_, *T*_*I*_, and *T*_*H*_ denote *recognition time*, *involvement time*, and *handling time*, respectively. During baseline trials it was assumed *T*_*R*_ = *0*.

During discrimination, participants often manipulated multiple test-objects before finally making a selection. The number of test-objects manipulated was recorded and defined as “encounters”. This total included the final selected test-object. Any trials that were invalid were excluded from the analysis, such as if the participant stopped to ask a question, or did not correctly follow the testing protocol.

### Data analysis

The block and spring test results were analyzed separately. *Accuracy* (A) was calculated as the number of *correct* test-objects a participant selected divided by the *total* number of test-objects a participant selected. Three time-duration measures were computed that broke down the average time spent in each of the foraging phases: *search time* (*T*_*S*_), *recognition time* (*T*_*R*_), and *handling time* (*T*_*H*_).

In order to determine the decisions that participants made when they were rejecting test-objects, we derived *false positive* (*alpha*, α) and *false negative* (*beta*, β) *error rates* using the number of test-objects participants encountered and Bayes’ Theorem. We used Bayes’ Theorem to statistically infer the rate at which participants made *alpha* and *beta* errors. Empirically determining these error rates would have required the stiffness of every individual test-object that the participant interacted with (picked up, assessed, and returned to the box) to have been known. With respect to the video analysis, the test-objects were not visually distinguishable which prevented us from empirically determining the stiffness of the test-objects that were rejected. Without knowing the stiffnesses of all the test-objects that were interacted with, Bayes’ Theorem allowed us to make a strong statistical inference about which blocks were being rejected. *Alpha* quantified the probability of the participant incorrectly selecting a non-target test-object given that the test-object in hand was a non-target object (false positive, see: Eqn. 2). It is important to note that *alpha* is different from the proportion of wrongly selected items. Conversely, *beta* quantified the probability of the participant incorrectly rejecting a target test-object given that the test-object in hand was a target object (false negative, see: Eqn. 3).

2 $$\alpha =P(s|\,\sim \,t)=\frac{P(\,\sim \,t|s)\ast P(s)}{P(\,\sim \,t)}$$

and

3 $$\beta =P(\,\sim \,s|t)=1-P(s|t)=1-\frac{P(t|s)\ast P(s)}{P(t)}$$

were calculated using the standard format of Bayes’ Theorem, where *P*(*s*) was the probability of selecting any given test-object (see: Eqn. 4), found by dividing the number of test-objects the participants were required to select (100) by the number of test-objects they encountered (*N*),

4 $$P(s)=\frac{100}{N},$$

and *P*(*t*) was the prior probability that any given test-object was the target stiffness, a foreknown quantity based on the concentration of each test-object stiffness. Tilde (~) is used to indicate negation. At the start of a trial, *P*(*t*) = 1/3, but as the participant removed test-objects, this quantity would increase or decrease as incorrect or correct test-objects were selected, respectively. We therefore used a corrected average *P*(*t*) value using each participant’s *accuracy* to determine the average concentration of correct test-objects remaining after each selection they made. A summary of all the video-extracted and calculated outcome measures are provided in Table 1. The datasets generated and/or analyzed during the current study are available from the corresponding author on request.

**Table 1:** A summary table of experimental measures.

(a) Measures extracted from video footage				
Measure	Symbol	Definition	Units	
Search time	*T*_*S*_	Time spent searching for to-be-selected test-object.	seconds	
Involvement time	*T*_*I*_	Time spent interacting with the to-be-selected test-object.	seconds	
Handling time	*T*_*H*_	Time spent transferring and releasing the selected test-object.	seconds	
Encounters	*N*	Number of test-object manipulated in a foraging cycle.	integer value	
(**b**) **Calculated measures**				
**Measure**	**Symbol**	**Definition**	**Units**	**Eqn**. **number**
Recognition time	*T*_*R*_	Time spent extracting sensory information to inform decision making.	seconds	(1)
Accuracy	*A*	The ratio of correctly selected test-objects to total test-objects selected.	% correct	n/a
Alpha error rate	*α*	The probability of incorrectly selecting a test-object (false positive).	probability	(2)
Beta error rate	*β*	The probability of incorrectly rejecting a correct test-object (false negative).	probability	(3)

(a) The measures extracted through frame-by-frame analysis of experimental video footage. (b) The measures derived through calculations of experimental data. Table categories provide: the symbolic annotation of each measure as used in the equations of the methods section, a general definition, the units of each measure, and the corresponding equation describing the derivation of each measure (where applicable).

### Statistical analyses

Comparisons and correlations across outcome metrics, stiffnesses, and test-objects were performed using bonferonni-corrected paired t-tests and Pearson’s correlation coefficients, respectively. P values less than or equal to 0.05 were considered significant.

## Results

**Figure 4:**
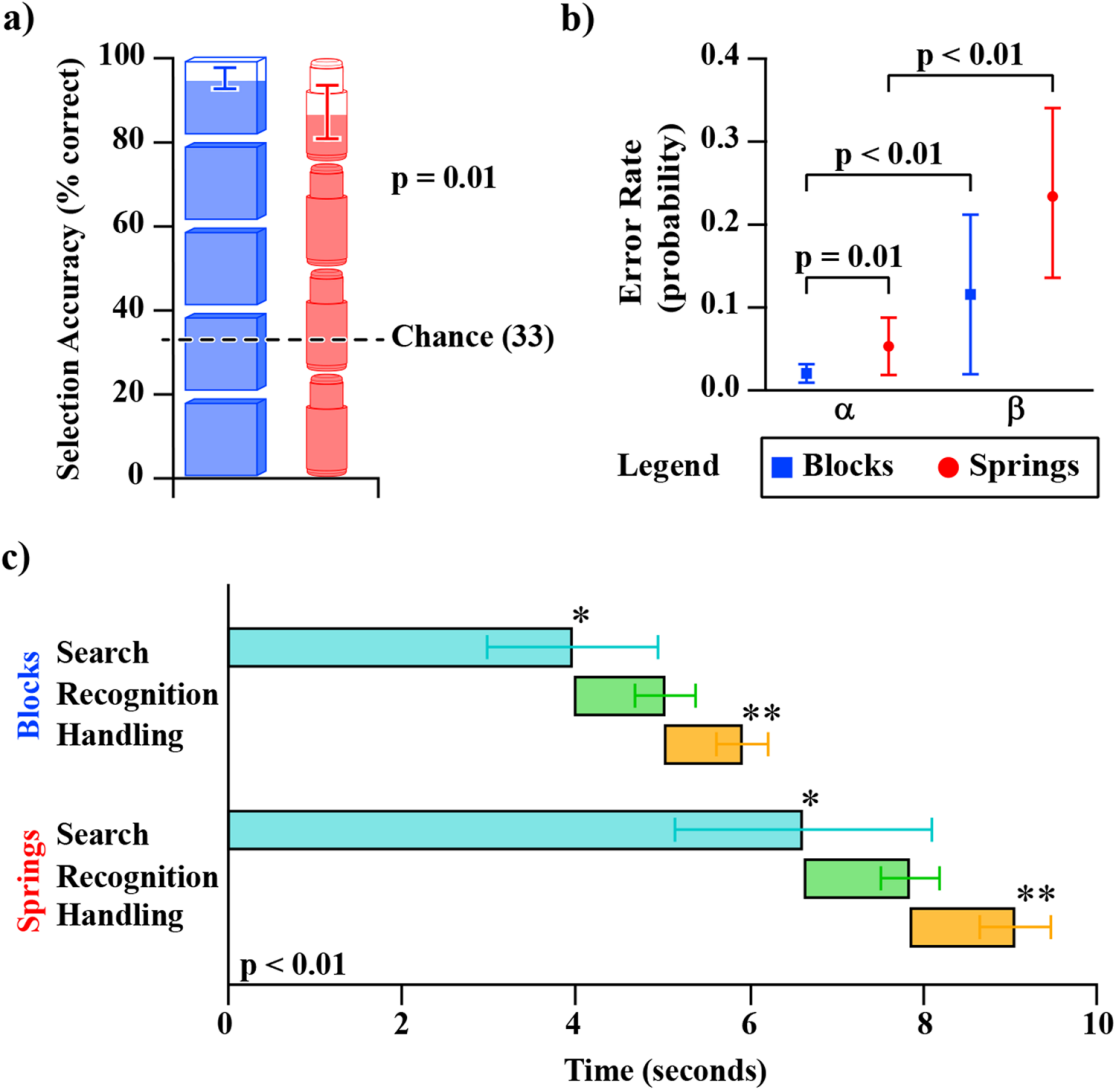
A breakdown of the six metrics collected during testing, for both blocks (blue) and springs (red). Panel (a) shows the average of all participants’ selection accuracies for both blocks and springs. The fill of the object segments represents the average number of correct objects the participants identified per trial (out of five blocks or four spring cells), whereas the y-axis indicates the percentage of correctly selected objects that the participants identified from all trials (as a percentage). Panel (b) shows average Type I error rate (α) and Type II error rate (β) of all participants. P-value indicates significantly lower error rates. Panel (c) shows the average time participants spent in each foraging phase for both blocks and springs. The staggered bar plots represent different foraging phases and correspond to the labels on the left. The sum of each set of three bars represents the average total foraging time per respective test-object. Note that the error bars describe the variability of each foraging phase independently, and are not additive. P-value indicates significance level of both * (the top bars) and ** (the bottom bars). All error bars represent ±1 standard deviation of the respective plotted variable.

**Figure 5:**
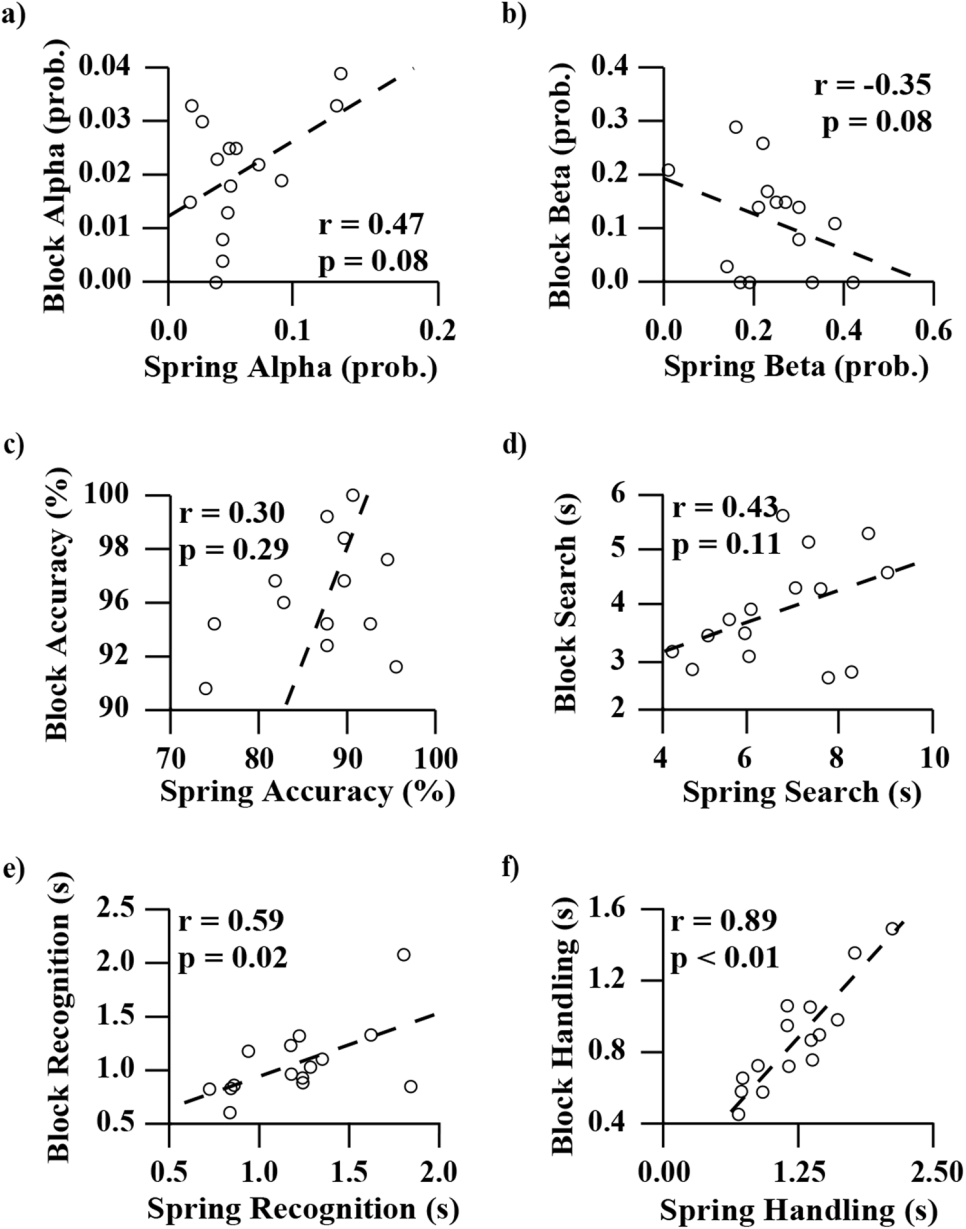
Correlations between block and spring performance for the six metrics collected during the test. Block performance is shown on the y-axes and spring performance is shown on the x-axes. Axes are scaled to include all data and maximize viewing area. Each marker indicates the block and spring performance of a different participant. The dotted lines are the trend lines. The correlation coefficient (r) and significance level of the correlation is labelled for each plot. The two plots at the bottom (Panels (e and f)) show significant correlations between blocks and springs for those for metrics. The label “prob.” denotes probability.

Our task and test-objects were designed to differentially prioritize the role of tactile and proprioceptive feedback in informing discrimination decisions^1^. Therefore, we compared performance scores (Fig. 4) and correlations (Fig. 5) in averaged and individual participant data, respectively, to characterize how this sensory-prioritization propagated throughout our measures. It was found that participants performed significantly better on 4 of the 6 block measures relative to the spring measures (Bonferonni-corrected paired t-tests, all p-values ≤ 0.01, Fig. 4). The block test scores demonstrated significantly higher *accuracy* (87.2% to 95.3%, p = 0.01), *search times* (6.61 s to 3.97 s, p < 0.01), and *handling times* (1.21 s to 1.06 s, p < 0.01) when compared to the spring test results. *Beta error rate* and *recognition time* were the only 2 measures demonstrating no statistical differences (p = 0.09 and p = 0.47, respectively). Strong and significant correlations were present across the two tasks for both *recognition* and *handling times* (r = 0.59 and r = 0.89, p = 0.02 and p < 0.01, respectively). *Search time*, which made up the majority of the time each participant spent foraging (Fig. 4c), had a moderate, non-significant correlation between the two tests (r = 0.43 and p = 0.11). *Accuracy*, *alpha error rate*, and *beta error rate* demonstrated weak correlations (Fig. 5).

**Figure 6:**
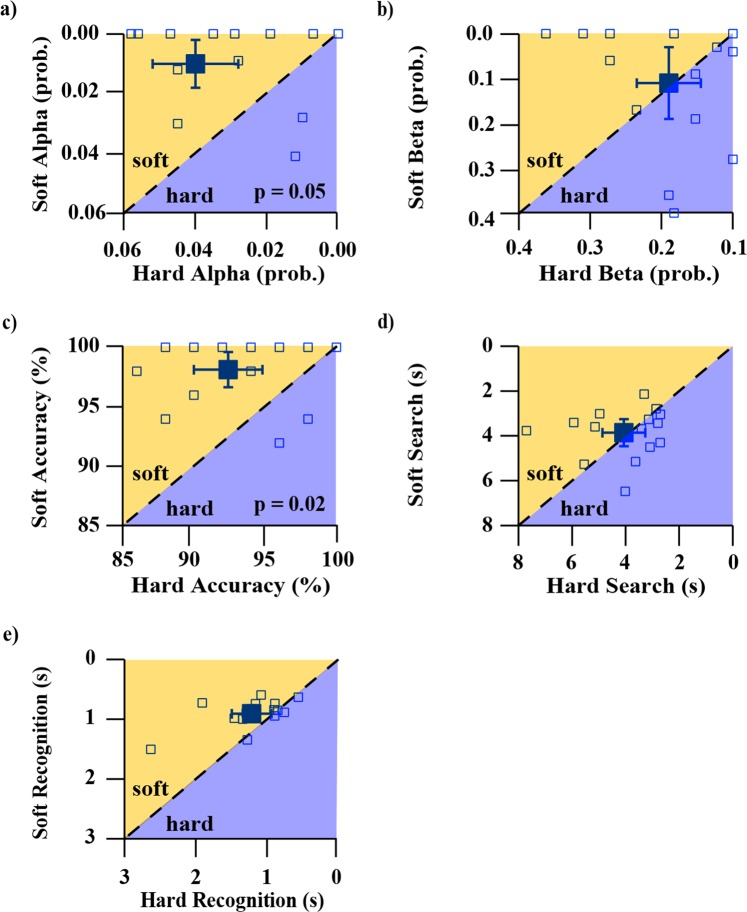
Soft blocks vs. hard blocks performance for five different metrics. All y-axes represent soft performance and all x-axes represent hard performance. For all panels, axes have been scaled/reversed such that better performance is to the top and right. Each open square marker represents a different participant. Larger filled square markers represent the average of all participants and the error bars represent 95% confidence intervals of the respective averaged data. The dotted 1:1 line in each plot represents equal performance between soft and hard blocks. Markers that lie above the dotted 1:1 line in the shaded orange region labelled *soft* indicate that participant performance for that metric was better for soft blocks, whereas markers that lie below the dotted 1:1 line in the shaded purple region labelled *hard* indicate that participant performance for that metric was better for hard blocks. Subplots 6a and 6c show statistically better performance for soft blocks than hard blocks, with labelled p-values indicating the significance level. The label “prob.” denotes probability.

**Figure 7:**
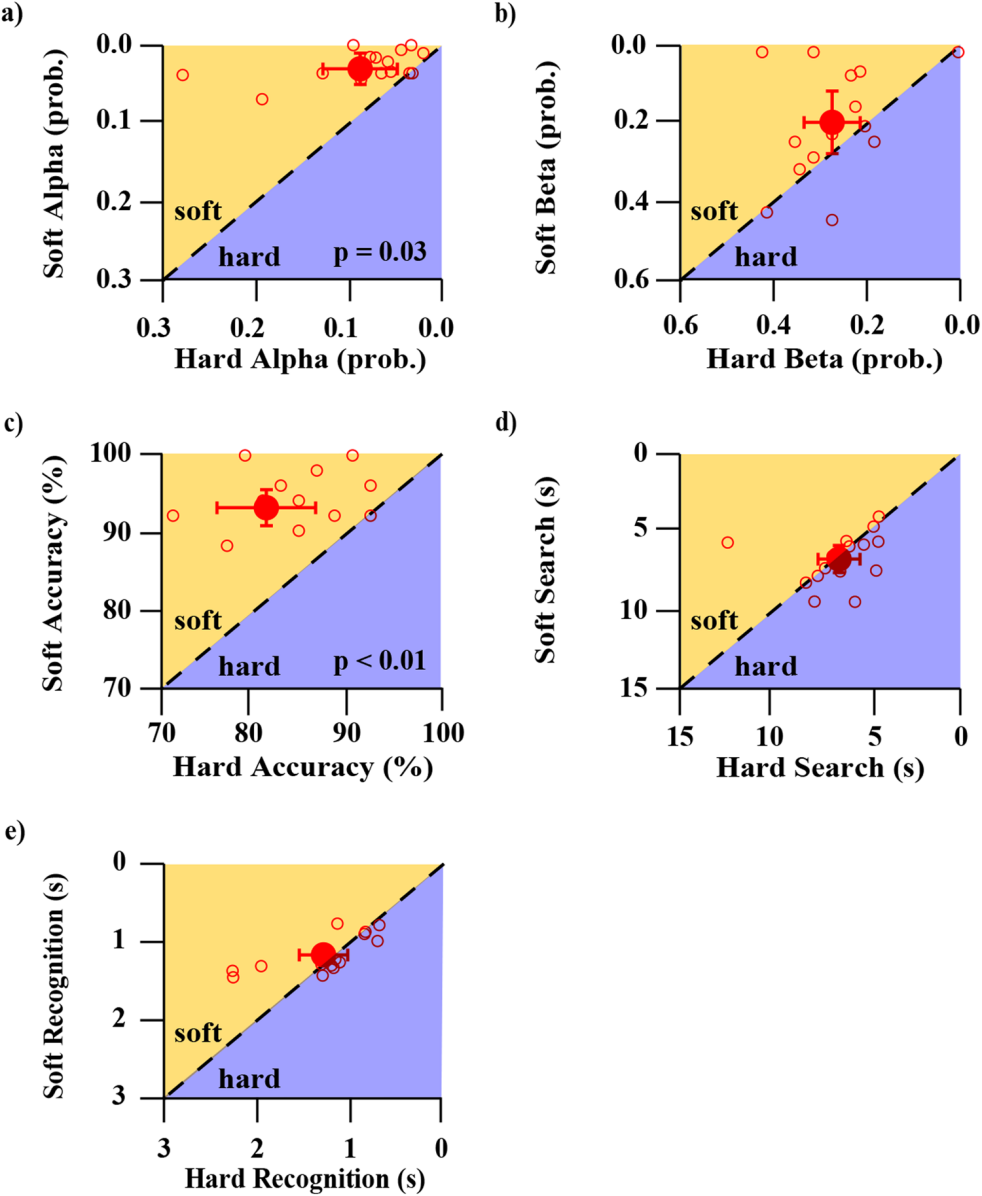
Soft springs vs. hard springs performance for five different metrics. All y-axes represent soft performance and all x-axes represent hard performance. For all panels, axes have been scaled/reversed such that better performance is to the top and right. Open circle markers each represent a different participant. Larger filled circle markers represent the average of all participants and error bars represent 95% confidence intervals of the respective averaged data. The dotted 1:1 line in each plot represents equal performance between soft and hard springs. Markers that lie above the dotted 1:1 line in the shaded orange region labelled *soft* indicate that participant performance for that metric was better for soft springs, whereas markers that lie below the dotted 1:1 line in the shaded purple region labelled *hard* indicate that participant performance for that metric was better for hard springs. Subplots 7a and 7c show statistically better performance for soft springs than hard springs, with labelled p-values indicating the significance level. The label “prob.” denotes probability.

Average participant performances between soft and hard objects were compared to quantify how physical changes in test-objects and the corresponding changes in sensory information manifested in the test scores. The non-linear scaling of test-object stiffnesses allowed us to assess how participants’ abilities to discriminate the objects changed across a range of stiffnesses, as well as validate our tests’ abilities to capture those changes in discriminatory ability. Soft-object versus hard-object comparisons were performed for the block test and spring test separately. On average, participants’ scores were improved when foraging for soft objects in two of the outcome measures: accuracy and alpha error rate (Figs 6 and 7); this was present in both the block and the spring tests (all p-values ≤ 0.05 using Bonferonni-corrected paired t-tests). The remaining measures: search time, recognition time, and beta error rate did not demonstrate significant differences in the performance during discrimination of hard and soft objects.

**Figure 8:**
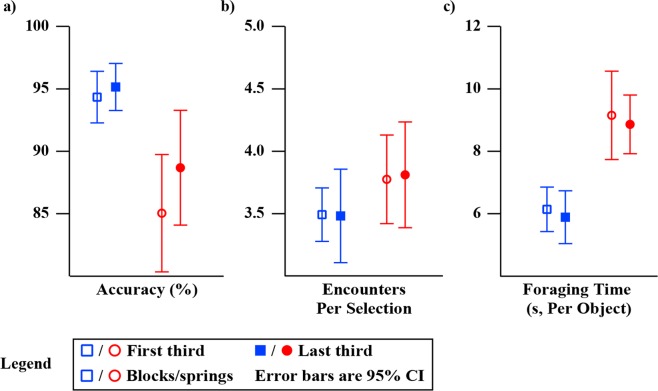
Group mean performance during the first third and the last third of the experiment. Group mean performance was examined for accuracy, encounters per selection, and foraging time. The block test performance is shown as blue squares and the spring test performance is shown as red squares. Empty markers represent performance during the first third of the test, which was approximately 33 trials, and filled markers represent performance during the last third of the test (also 33 trials). Error bars represent 95% confidence intervals of the respective plotted variables. Axes are scaled to maximize viewing area.

We expected no learning time or learning effects for our test as using one’s senses of touch and proprioception to determine an object’s stiffness is a common everyday task. To quantify learning effects, we examined the average performance of each of our participants during the first third (33 trials) and last third (33 trials) of the block and the spring tests (Fig. 8). *Accuracy*, number of encounters per object selection, and average foraging time were specifically evaluated as every remaining measure was derived from these three variables. No significant differences (using paired t-tests) were detected for either the block or the spring tests.

**Figure 9:**
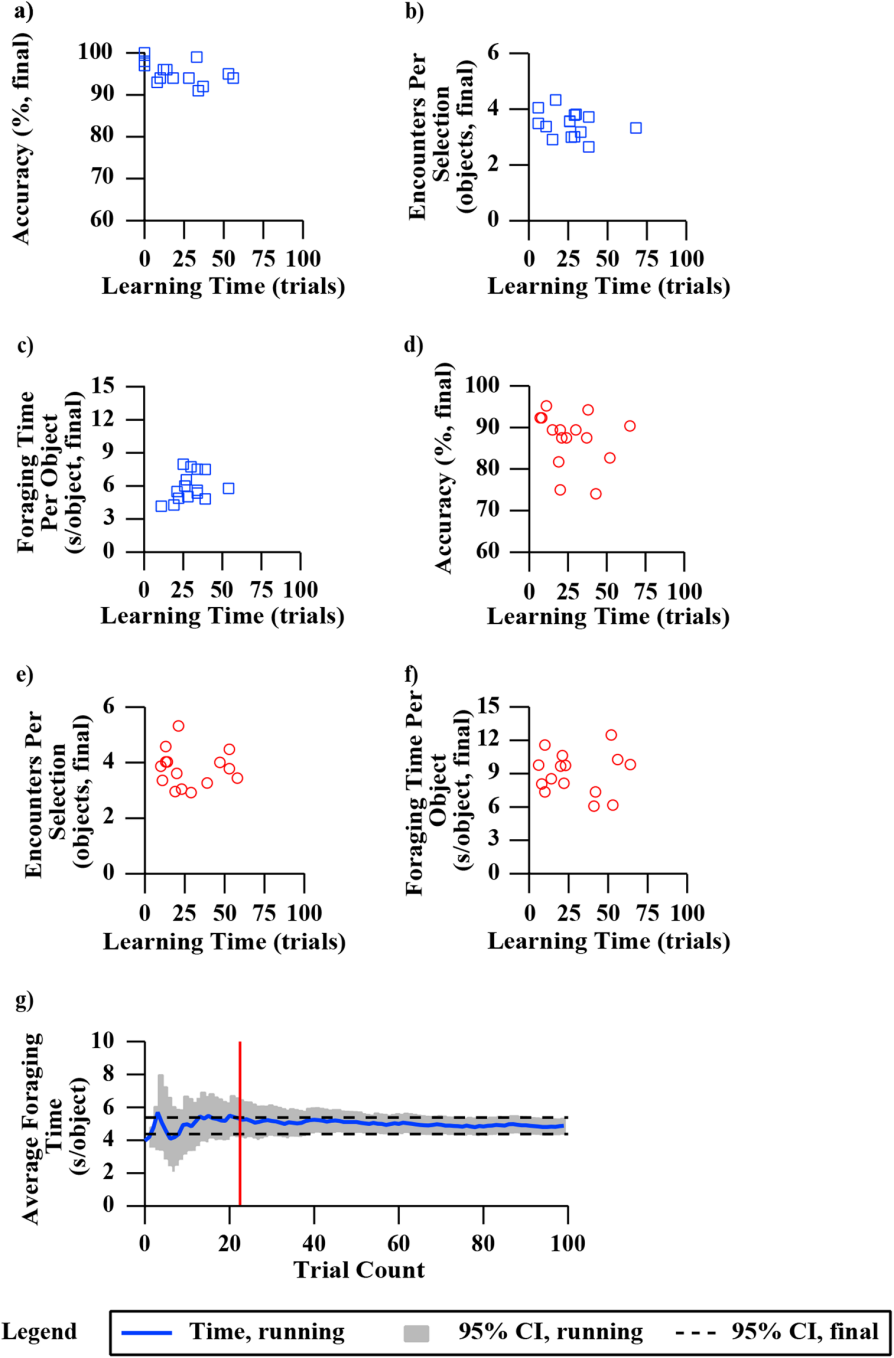
Comparing each participant’s learning time to their overall performance for blocks and springs. Learning time was measured in number of trials, and performance was examined in three different measures (accuracy, encounters per selection, and foraging time per object). Blue blocks represent participant data from the block test (**a**–**c**) and red circles represent participant data from the spring test (**d**–**f**). For (**a**–**f**), the x-axis shows the number of trials spent “learning”, that is, the number of trials it took each participant for their performance to stabilize within the 95% confidence intervals of their final performance. (**g**) Shows an example participant to illustrate how learning time was determined. The number of trials for the participant’s running average performance to enter and stay within the 95% confidence intervals of their final performance was considered to be the number of trials spent “learning”. Stable performance for this participant is indicated by the red vertical line.

Additionally, we explored the relationships between learning effects and overall performance by evaluating correlations between the number of trials a participant spent “learning” and their final scores (Fig. 9). The running average score (starting with trial one) was used, and “learning time” was defined as the number of trials required for each participant’s running average to enter and stay within the bounds of their *final* 95% confidence interval (Fig. 9g). Each participant’s learning time was compared against their final overall performance in *accuracy*, encounters per selection, and foraging time per object (Fig. 9a–f). No correlations between learning time and overall performance for any of the three measures in either the block or the spring tests were found.

## Discussion

The objective of this work was to develop a psychophysical touch and proprioception measure that would allow us to investigate sensorimotor behavior and strategy of participants as they performed a sensory discrimination task. The development of our metric was guided by five key criteria. (*1*) *Minimal rules and instructions:* The acts of selecting, discriminating, and transferring test-objects were unconstrained, and participants were given an initial practice period to organically arrive at their preferred sensorimotor strategies. (*2*) *Sensitivity to sensorimotor strategies:* Our sensory discrimination tasks provided multiple outcome measures sensitive to sensorimotor strategies. For example, if a participant were overly cautious in their discrimination we would anticipate increased *search time* and increased *beta error rate*, or if a participant were simply guessing and transferring objects as quickly as possible, this would present as an *accuracy* close to chance with reduced *search* and *recognition times*. (*3*) *Sensitivity to the effects of sensory feedback:* Our tasks were specifically designed to evaluate one’s ability to make discrimination choices informed through cutaneous and proprioceptive sensory information. (*4*) *The elimination of ceiling effects:* Ceiling effects may occur in clinical tests when patient performance improves to the point that the test is insufficiently difficult to measure their ability, and thus the test can no longer detect improvements in their condition. To the detriment of allowing comparisons across populations, often a test will stratify users by providing tasks that are insufficiently challenging for able-bodied, yet too difficult for the sensorimotor impaired. Our task provides a suite of metrics, including time-based measures, which scale in our application to accommodate all participant populations and their abilities. (*5*) *Multiple outcome scores rather than a single* “*good*/*bad*” *or completion-time score:* Our utilization of an OFT cryptic prey style video analysis provided a variety of measures to characterize foraging and decision making behaviors. Specifically, we extracted four quantitative measure from experimental data to directly characterize task performance. Additionally, error analyses were performed, creating predictive models of the types of errors participants were most likely to make. Together, these outcomes allow for a nuanced description of sensorimotor performance.

Although the acts of manipulating, discriminating, and transferring a test-object are relatively straightforward, information is available that enables measures reflecting three primary aspects of performance: motor control, sensory feedback, and strategy (Table 2). ***Recognition time*** is almost exclusively a function of sensory feedback, as it accounts for the time that the participants spent extracting sensory information from a test-object and making a discrimination choice. ***Accuracy*** is a measure of how successful a participant’s sensory system was in providing relevant information to guide the selection of a test-object. *Accuracy* is also affected by the strategic decisions of the participant, such as the degree of caution used when selecting objects. ***Handling time*** is calculated from baseline trials, which consist of only the grasp and transfer of the selected test-objects, and therefore is primarily a function of the participant’s motor control. This phase is nearly identical to the traditional Box and Block test^46^: during baseline trials, participants are instructed to move the test-objects one at a time, over the center partition, into the other compartment as quickly as possible. ***Search time*** is dependent on the sensory and motor systems as the participant is required to navigate through the experimental space while manipulating test-objects, using sensory information to reject non-target objects, and locate a target object. A participant might strategize during *search time* by minimizing the re-visiting of non-target test-objects or by searching in “hot spots”. The ***alpha error rate*** (false positive) describes the probability that the participant will mistakenly select a non-target object each time one is encountered. *Alpha error rates* are minimized through the use of sensory information and strategic decisions, such as weighing the discrimination certainty against the time cost of finding a new object. ***Beta error rate*** (false negative), which describes the probability that the participant will mistakenly reject a target object each time one is encountered, is influenced by both sensory feedback and strategy in a similar manner.

**Table 2:** The sensitivity of individual performance indicators and their sensitivity to sensory, motor, or strategic aspects of upper limb control.

Outcome measure	Sensory	Motor	Strategy
Recognition time	✔		
Accuracy	✔		✔
Alpha error rate	✔		✔
Beta error rate	✔		✔
Handling time		✔	
Search time	✔	✔	✔

Comparing participants’ results between the block and spring cell tasks allowed us to describe how participant strategy and performance changed based on the sensory modalities with which they presumably interacted, and characterize which aspects of performance translated across the test variants. Previous work by Srinivasan and LaMotte revealed that the sensory modalities needed to discriminate objects of different stiffnesses was in part dependent on the surface deformability of those objects. Specifically, compliant objects with deformable surfaces (rubber specimens) required only tactile sense to discriminate, whereas compliant objects with non-deformable surfaces (spring cells) required both cutaneous pressure and proprioception to discriminate^1^. We designed our tasks using their findings as a guide so that the two variants of our task, blocks and spring cells, would each prioritize a different sensory modality: cutaneous pressure and proprioception, respectively. In our analyses, it was found that the average *accuracy* of the block test was significantly higher than the spring test (Fig. 4a), with the average foraging time being significantly less (Fig. 4c). Additionally, the block and the spring test *accuracy* scores did not demonstrate correlation. This implies that different *accuracy* was being achieved across the differentially prioritized sensory modalities, and the magnitude of these differences were individual to each participant. These findings are paralleled by Srinivasan and LaMotte. In their experiments, participants also made stiffness discriminations about rubber test-objects and spring cells and differences in performance were attributed to presence of cutaneous touch and proprioception, respectively^1^. However, in their work, participant finger indentation parameters (e.g., joint angles, loading velocities, peak force contact, and specimen edge avoidance) were carefully controlled, and discrimination was tested under both active touch (with kinesthesia) and passive touch (without kinesthesia)^1^. This is in contrast to our study where participants were not constrained in how they manipulated the test-objects. Despite this, our data, combined with the findings of Srinivasan and LaMotte, support the idea that both tests are unique sensory tasks evaluating two separate sensory aspects of upper limb control.

*Recognition time* is a sensory-based value that represents the time required to extract and interpret sensory information, as well as make a decision. We found that regardless of the sensory modalities being prioritized in the block and the spring tests, the average *recognition times* did not significantly differ but instead demonstrated significant correlation. This suggests that the sensory content (cutaneous-force, or proprioception and cutaneous-force) does not significantly influence this time, and/or any non-significant differences are scalable across individual participant performances. When foraging for cryptic prey, there is an optimal tradeoff between search rate and the probability of prey detection. Slow searching may provide improved prey detection but requires more time, and searching faster allows more area to be investigated but may detect less prey; thus, there is a tipping point where neither alternative will further increase the overall rate of prey capture^48^. In our case, search rate and probability of prey detection are both heavily influenced by *recognition time*. Therefore, the average *recognition time* that we found (approximately 1.1 seconds [Fig. 4c]) likely represents the optimized duration for participants to spend engaging with sensory information, regardless of the sensory content; any further time would yield diminishing returns on the quality of the discrimination decision.

Different *handling time* durations may be expected between the tasks as the two test-objects are different shapes and require different methods for grasping and handling. Indeed, we found that participant *handling times* measured from the block task were significantly different than those measured in the spring task. We also found that participant *handling times* between the block and spring tasks were strongly correlated (Figs 4c and 5f), i.e., participants who transferred objects quickly in one test were likely to be quick in the other. *Handling time* is primarily influenced by motor control and therefore a marker for the motor capabilities of the person being tested. In our experiments, participants were effectively using identical motor control systems (with arguably similar motor capabilities): their physiological hand and arm. We suggest that in this case, differences in participants’ *handling times* could also be thought of as a proxy for level of engagement with the task.

We examined the participants’ test results for learning effects to understand how sensory feedback modality influenced the rate at which participants optimized the foraging discrimination task. Additionally, we quantified correlations between how quickly a participant learned and their overall task performance. This provided insight into how the “slowness” or “quickness” of a participant’s learning may be related to their overall task ability, e.g., are fast learners better at the task? No significant difference was demonstrated in average performance during the first and last third of testing, in *accuracy*, encounters per selection, or foraging time (Fig. 8). This finding supports that participant performances were stable over the duration of testing. Furthermore, the size of the final 95% confidence intervals suggest that this likely stems from minimal changes in mean values rather than large variances in scores. Figure 9 highlights the number of trials prior to participants converging on stable performance (maintaining scores within their final 95% confidence interval). No statistically significant correlations were found between the number of trials to converge and performance. In other words, how quickly one learns the task and adopts a sensorimotor strategy does not appear to influence their ability to perform the task. As a group, participants typically demonstrated convergence quickly within the first third of the trials.

**Figure 10:**
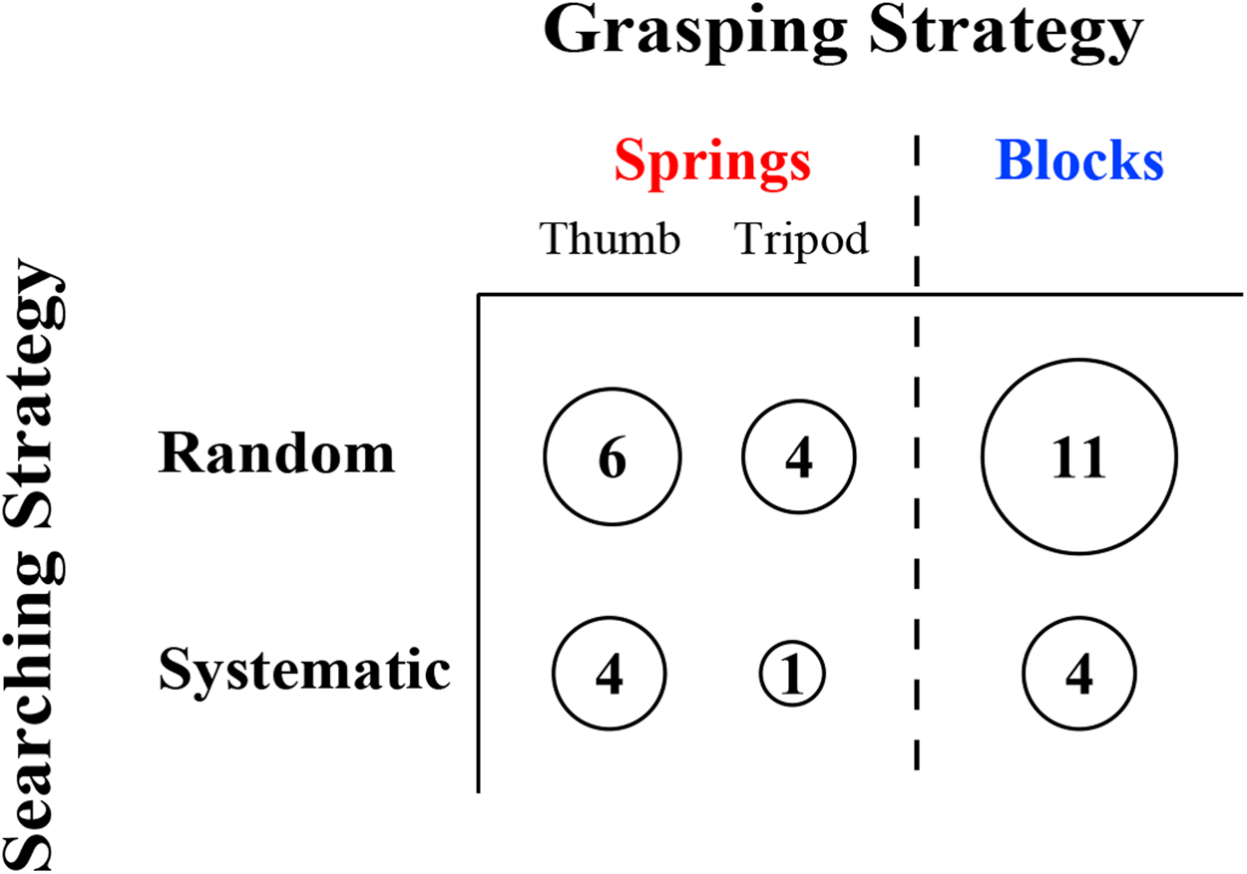
Observed proportions of participants using different grasping and searching strategies. Participants were classified into two strategies of grasping (top: thumb versus tripod) and two strategies of searching (left: random versus systematic). The total number of participants using each searching strategy or each grasping strategy is shown by adding the numbers across each row or each column, respectively. The number of participants using particular combinations of grasp and search strategies can be seen by looking at specific cells (e.g., the number of people who searched systematically with a tripod grasp is shown in the bottom center cell.)

Our tasks were specifically designed to be unstructured in the way participants engaged with, and explored test-objects; yet, during the experiments, we observed some participants adopting organized strategic behaviors. As our analyses did not explicitly quantify these behaviors, we completed an additional observation-based analysis to capture the way participants *searched* and *grasped* test-objects (Fig. 10). Specifically, when searching, some participants engaged in more “systematic” search styles, such as organizing rejected test-objects to separate them from potential target objects, and/or working exhaustively from one area of the box to another. This is in contrast to more “random” search strategies, where participants placed rejected test-objects in their original location or randomly around the box, and switched their search area erratically. We classified participants as systematic or random searchers based on how frequently and deliberately they organized rejected test-objects, and how likely they were to exhaustively search test-objects by location. Although systematic strategy was observed in some participants, the majority of participants fell into the random searchers category. This finding is consistent with other biological foragers, in which semi-random search strategies are employed and may be described through stochastic mathematical models such as Levy flight^49^. Importantly, traditional OFT models assume a memoryless random search phase, and in our data, no difference in performance was observed between participants performing random and strategic-sorting search strategies, supporting that the memoryless assumption stands for our analyses. We also observed *grasping* strategies during the spring test. Participants fell into one of two categories: thumb grasping and tripod grasping, as described in Methods. We classified all participants into these searching and grasping strategies (Fig. 10). The strategy one adopts to manipulate the spring cells may have two possible explanations: (1) participants may have selected their grasping strategy for motor control reasons, e.g., they selected a method which allowed them to grasp the spring cells quickly, comfortably, or in a way that minimized muscular exertion or fatigue, (2) a participant’s grasping strategy may have been selected on a sensory discrimination basis, e.g., they determined that their chosen grasping method allowed them to most accurately and quickly collect sensory information. Significant differences between thumb grasp and tripod grasp strategies were not found in any of our performance measures (data not shown), suggesting that either the effect of grasping strategy was too small to detect, or grasping strategy was selected based on some other unknown variable.

In OFT studies that examine the optimal diet of foragers, the long-term rate of caloric gain and profitability (the value of an encountered prey item) play a fundamental role in predicting the prey items that are included as part of the forager’s optimal diet. The optimal diet model suggests that the most profitable prey item is always included in the forager’s diet. From this point, the inclusion of prey items into the diet is considered in order of descending profitability, and a prey item is included in the forager’s diet *only* if the prey’s profitability is greater than the forager’s net rate of food intake^50^. Although this is a cursory overview of the optimal diet model, the relevant point pertaining to the present study is that rate of net gain and profitability have been demonstrated to be predictive in the decision-making of biological foragers. The values of rate of gain and profitability may have alternatives in the present study, and these values may potentially be useful with respect to the future goals of our sensorimotor task: characterizing sensory-impaired populations and sensory restoration interventions.

First, it is important to consider the concepts of prey reward and foraging accuracy. Generally, the forager’s reward from the prey is the prey’s (net) caloric content, whereas accuracy typically refers to the forager’s detection rate of the prey from the environment. In the present study, these are not distinct concepts; participants are only rewarded for detecting correct test-objects, (e.g., if soft test-objects are the target, medium and hard objects are both equally valueless). Any assigned reward value to the test-objects would simply be a scaling factor, and we therefore consider the reward of foraging to be interchangeable with the accuracy of foraging in our task. This departure from classical foraging theory affects the interpretation of foraging metrics, but better suits the long-term goals of our sensorimotor task.

The mathematical rate of gain of a forager’s diet is the long-term net caloric intake divided by total foraging time (described in units of caloric gain per unit time). In contrast, we calculate a modified version of rate of gain by dividing the number of blocks correctly selected by total task time (see: Eqn. 5). We refer to this quantity as *efficiency*. Although, this calculation has exceptions from classical rate of gain, we anticipate it may be useful to describe the effectiveness of therapeutic interventions in sensorimotor-impaired populations. For example, comparing two or more treatments might reveal differential improvements in sensory feedback and motor function, which may be captured through the measures of accuracy and foraging time, respectively. This hypothetical tradeoff between accuracy and speed could be quantified with *efficiency*. This would help to determine which intervention may provide the greatest benefit, just as rate of gain is used in the optimal diet model to predict which prey would benefit a forager’s diet.

Profitability is an essential quantity for a forager to (instinctively) consider when deciding whether or not to pursue a prey item. Profitability is calculated by dividing prey net caloric content by handling time, describing the relationship between an expected gain and the time taken to achieve it. In contrast, we calculate a modified version of profitability by dividing accuracy by recognition time (see Eqn. 7). We refer to this ratio as *discrimination efficiency*. The expected gain of each test-object is a function of the participant’s ability to correctly discriminate the test-object (accuracy). The time taken to achieve that gain is the time spent making the discrimination decision (recognition time). While this is similar to profitability, there are important differences. Profitability is a *prospective property of a prey item* that is known to the forager upon encountering that prey item, and is therefore a strong predictor of the forager’s decision-making. Conversely, *discrimination efficiency restrospectively describes the quality of the participant*’*s decisions* when selecting test-objects. A participant is not able to determine the value of a test-object (the likelihood that it is a target) until a time cost has already been incurred. Therefore, *discrimination efficiency* would not be a predictor of a participant’s decision-making when selecting test-objects. However, *discrimination efficiency* provides a meaningful way to quantify the performance of a sensory system. If two sensory restoration interventions are compared, they may provide different benefits to the quality of sensory information received versus how quickly that sensory information is useful to the patient. *Discrimination efficiency* provides a method to determine if tradeoffs between speed and accuracy are beneficial in a way that resembles how biological foragers make decisions about the balance between payoff and time investment.

Because the present study examined the performance of only able-bodied participants with intact sensorimotor systems, the measures of *efficiency* and *discrimination efficiency* would be expected to simply show trends in performance, rather than quantifying tradeoffs between alternative interventions as described above. However, able-bodied performance data would provide a useful frame-of-reference for contextualizing performance improvements in sensorimotor impaired populations, so those able-bodied data are included here. Each participant’s e*fficiency* (*E*, Eqn. 5) was calculated as

5 $$E=\frac{A}{{T}_{S}+{T}_{R}+{T}_{H}},$$

where *A* was their accuracy, and *A* was divided by the sum of their search time, recognition time, and handling time averages (*T*_*S*_, *T*_*R*_, and *T*_*H*_, respectively). Each participant’s *discrimination efficiency* was calculated using an *adjusted accuracy* (*A*’, Eqn. 6)

6 $$A^{\prime} =A-\frac{1}{2}(1-A),$$

so that an *accuracy* of 33% would yield an *adjusted accuracy* of 0. *Adjusted accuracy* was specifically used to accommodate for scoring based on chance, i.e., if a participant was unable to discriminate the test-objects above the threshold of guessing (33% *accuracy*), they would receive an *adjusted accuracy* and *discrimination efficiency* score of zero (reflecting the participant’s discrimination strategy relative guessing). *Discrimination efficiency* (*D*, Eqn. 7) was therefore calculated as

7 $$D=\frac{A^{\prime} }{{T}_{R}}.$$

**Figure 11:**
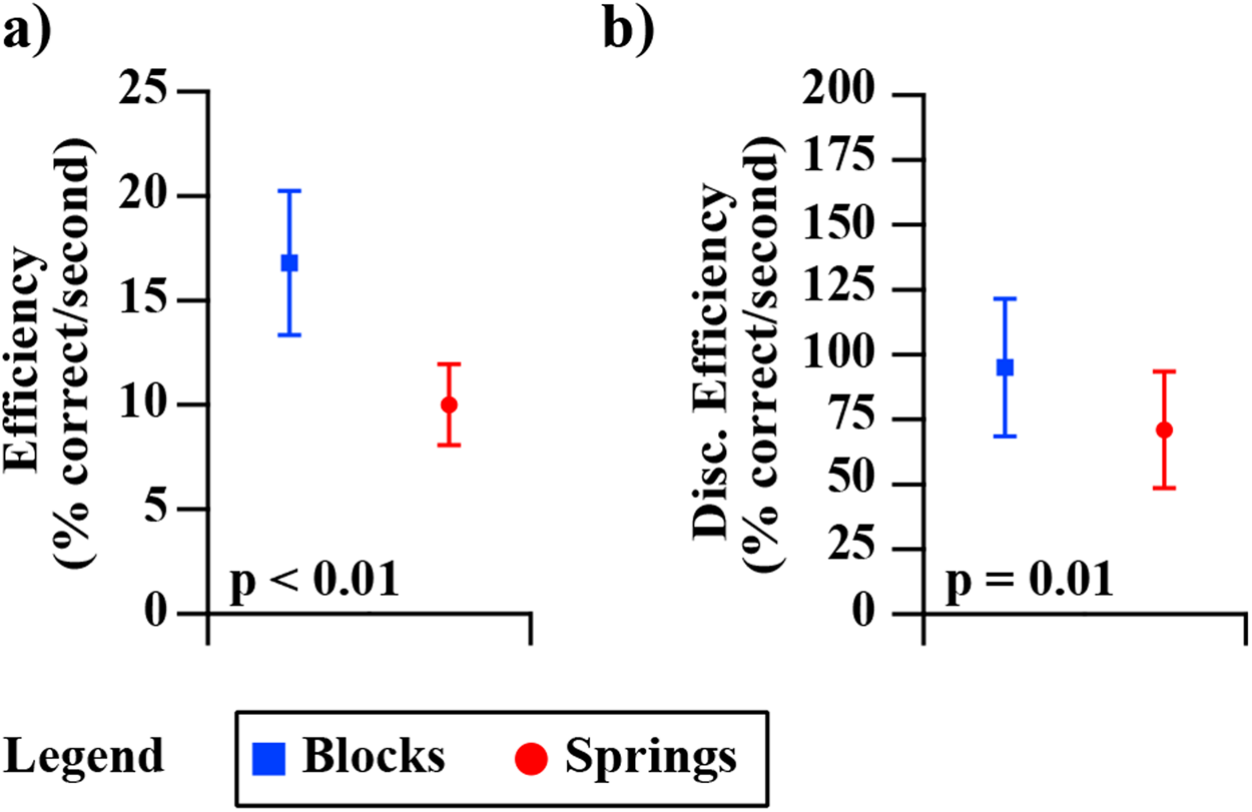
Group-mean performance in *efficiency* and *discrimination efficiency* for blocks (blue) and springs (red). Panel (a) shows average *efficiency* of all participants. P-value indicates significantly higher *efficiency* in the block test. Panel (b) shows the average *discrimination efficiency* of all participants for blocks (blue) and springs (red). P-value indicates significant difference in group means.

**Figure 12:**
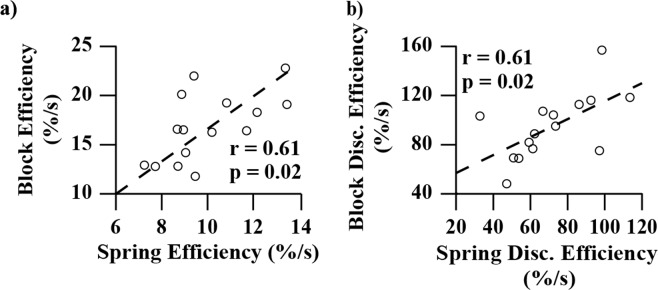
Correlation between block and spring performance for *efficiency* and *discrimination efficiency*. Block performance is shown on the y-axes and spring performance is shown on the x-axes. Axes are scaled to include all data and maximize viewing area. Each marker indicates the block and spring performance of a different participant. The dotted lines are the trend lines. The correlation coefficient (r) and significance level of the correlation is labelled for both plots. Both metrics showed significant correlation across the block and spring tasks.

**Figure 13:**
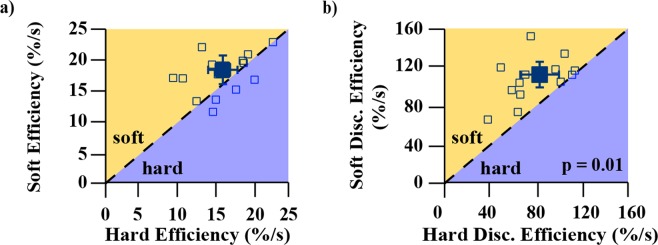
Soft blocks vs. hard blocks performance for *efficiency* and *discrimination efficiency* metrics. Both y-axes represent soft performance and both x-axes represent hard performance. For both panels, axes have been scaled such that better performance is to the top and right. Each open square marker represents a different participant. Larger filled square markers represent the average of all participants and the error bars represent 95% confidence intervals of the respective averaged data. The dotted 1:1 line in each plot represents equal performance between soft and hard blocks. Markers that lie above the dotted 1:1 line in the shaded orange region labelled *soft* indicate that participant performance for that metric was better for soft blocks, whereas markers that lie below the dotted 1:1 line in the shaded purple region labelled *hard* indicate that participant performance for that metric was better for hard blocks. Average participant *discrimination efficiency* (subplot 13b) was significantly higher for soft blocks compared to hard blocks

**Figure 14:**
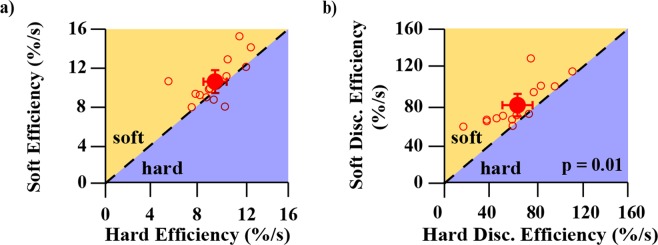
Soft springs vs. hard springs performance for *efficiency* and *discrimination efficiency* metrics. Both y-axes represent soft performance and both x-axes represent hard performance. For both panels, axes have been scaled such that better performance is to the top and right. Open circle markers each represent a different participant. Larger filled circle markers represent the average of all participants and error bars represent 95% confidence intervals of the respective averaged data. The dotted 1:1 line in each plot represents equal performance between soft and hard springs. Markers that lie above the dotted 1:1 line in the shaded orange region labelled *soft* indicate that participant performance for that metric was better for soft springs, whereas markers that lie below the dotted 1:1 line in the shaded purple region labelled *hard* indicate that participant performance for that metric was better for hard springs. Average participant *discrimination efficiency* (subplot 14b) was significantly higher for soft springs compared to hard springs.

We found that in the block task compared to the spring task, participants performed with greater *efficiency* (16.8%/s to 10.0%/s, p < 0.01) and *discrimination efficiency* (94.7%/s to 71.3%/s, p = 0.01) shown in Fig. 11. Additionally, *efficiency* as well as *discrimination efficiency* each strongly correlated across the two tasks (both r = 0.61 and p = 0.02), shown in Fig. 12. Comparing soft test-objects against hard test-objects revealed that there was not a significant effect of test-object stiffness on *efficiency* for either blocks or springs (Figs 13a and 14a). However, group-mean *discrimination efficiency* was significantly higher for soft compared to hard test-objects for both blocks and springs (Figs 13b and 14b), likely due to higher accuracy scores in soft test-objects (Figs 6c and 7c) while changes in recognition times were insignificant (Figs 6e and 7e).

While our task design and video analysis were influenced by OFT, we made several departures from classical OFT which are important to consider as they affect the interpretation of our results from an OFT perspective. First, accuracy (or prey detection) and prey value (e.g., calories) are conventionally separate quantities which together define a forager’s behavior^48^. In our task, we chose to assign value to correct discriminations only, as this allowed us to separate discrimination ability (e.g., accuracy) from foraging strategy (e.g., *alpha* and *beta* error rates). In contrast, if test-objects of different stiffnesses were differentially valued, then it would be unclear if the resulting accuracy and error rates of those objects were the result of an inability to discriminate them, or a strategic decision to ignore them in an attempt to improve foraging *efficiency*. The result of this decision was that a participant’s accuracy (the number of correct discriminations divided by the total foraging attempts) was synonymous with their foraging reward. Second, our adoption of accuracy as prey value carried over to the quantities of *efficiency* and *discrimination efficiency*. Conventionally, these quantities refer to the value of prey items being foraged for; that is, a prey item has a profitability value relevant to the forager, and this profitability value is the basis for modelling the forager’s decisions. In our case, we were not interested in describing the value of the prey items. Rather, our goal was to describe participants’ abilities to discriminate the test-objects, as well as characterize the time spent and decision-making which led to the discrimination decisions. Since our task design eliminated the aspect of prey choice, *efficiency* and *discrimination efficiency* described the value of the single target stiffness relative to its cryptic background (the distractor test-objects). In this way, *efficiency* and *discrimination efficiency* may also be thought of as describing the value of the participant’s discrimination ability and foraging strategy when foraging for the target stiffness (the only prey available to them).

At a fundamental level, decision-making in OFT is generally explained through the mechanisms of Marginal Value Theorem and the time opportunity cost of foraging^51,52^. When a forager encounters a prey, it must make a decision of whether to pursue that prey item, or ignore it and continue searching. This decision depends on the immediate reward of the prey item and the time opportunity cost of engaging with the prey item, the latter being defined by the forager’s average net energy intake. When a forager encounters a prey item, it must decide whether pursuing it or ignoring is a better use of its time, based on the reward it expects to receive from either option. This fundamental decision-making mechanism of foraging is also present in our stiffness discrimination task. Each test-object that a participant selects or rejects carries with it an associated opportunity cost. Each time a participant encounters a test-object, they inherently decide the probability that it is a target test-object (i.e., its value) and they know the amount of time they have spent thus far finding and identifying the test-object. This is the immediate value of the test-object. The participant must then make the decision to select it or reject it, and the forgone decision becomes the opportunity cost. The more confident the participant is that they have a correct test-object, the greater the opportunity cost of rejecting it (i.e., making a false negative error), as they will incur more time cost and receive diminishing gains in accuracy. Inversely, there is a tremendous opportunity cost associated with taking an incorrect test-object (i.e., a false positive error), as it provides 0 value, and in our task the number of foraging attempts allowed is explicitly limited; if an incorrect test-object is taken, the opportunity to find a correct one is permanently lost. Even at longer foraging times, there is still a great opportunity cost for taking an incorrect test-object as it provides no value. It would follow from the opportunity cost argument that participants in our task should bias towards false negative errors, while taking great care to avoid false positive errors, as false positive errors have large opportunity costs. We do in fact see this in the data, as for both the block and spring tasks, participants were significantly less likely to make false positive errors than false negative errors (Fig. 4b).

We developed a foraging style task, that assessed the strategy and performance of human participants completing sensory discriminations. Our data analysis was able to describe the strategies used by the participants, as well as identify overall performance trends of a normative population to characterize how humans engage with object search-and-acquisition in the context of a tactile and proprioceptive sensory discrimination task. We suggest that this task could be a valuable tool for studying touch and proprioception, such as understanding the impact of altered sensation and/or motor control in impaired populations or those using assistive technologies such as upper limb prostheses. Our task may also have potential to be adapted as a format for foraging theory studies as a test-bed or validation tool in applicable foraging models.
